# Diabetes Mellitus and Risk of Hepatocellular Carcinoma

**DOI:** 10.1155/2017/5202684

**Published:** 2017-12-12

**Authors:** Xu Li, Xiaocong Wang, Pujun Gao

**Affiliations:** ^1^Department of Hepatology, The First Hospital of Jilin University, Jilin University, No. 71 Xinmin Street, Changchun 130021, China; ^2^Department of Echocardiography, The First Hospital of Jilin University, Jilin University, No. 71 Xinmin Street, Changchun 130021, China

## Abstract

The occurrence of hepatocellular carcinoma (HCC) is two to three times higher in patients with diabetes mellitus (DM), the prevalence of which is increasing sharply worldwide. The purpose of this review was to describe clinical links between DM and HCC and potential biological mechanisms that may account for this association. We evaluated the role of potential pathways that could account for the development of HCC with different etiologies in the presence of DM. In addition, we also briefly discuss the potential effect of other factors such as type and dosage of antidiabetic medicines and duration of DM on HCC risk.

## 1. Introduction

Diabetes mellitus (DM), a metabolic disorder characterized by dysregulation of blood sugar and insulin [1–3], has an estimated global prevalence of approximately 9% and is expected to affect 300–400 million worldwide by 2030 [4–10]. In 1986, Lawson et al. reported a relationship between DM and hepatocellular carcinoma (HCC), which is the sixth most common cancer worldwide and accounts for 11% of cancer-related deaths [11]. Multiple observational studies from Europe, Asia, and North America and subsequent meta-analyses support the idea that DM and insulin resistance are independent risk factors for HCC [12–14]. The development of HCC is thought to be related to the proliferative effects of insulin and insulin-like growth factor 1, oncogenic effects of hyperglycemia, and inflammatory effects of obesity [15]. This relationship between DM and HCC is significant even after adjusting for detection bias and reverse causality [16]. In addition, the type and dosage of antidiabetic medication used [17] appear to affect the risk of HCC.

In this clinical review, we present the epidemiological evidence linking DM and HCC, discuss potential molecular mechanisms underlying the development of HCC with different etiologies in patients with DM, and describe the effects of antidiabetic medications and duration of DM on HCC risk.

## 2. Epidemiological Studies Linking DM and HCC

When DM was first investigated as a risk factor in cancer-related deaths, the causes of these two diseases were unknown [18]. In 1934, a study of 10,000 diabetic patients reported an association between pancreatic cancer and DM [19]. In 1991, a large population-based cohort study conducted by Adami et al. in Sweden (*n* = 51,008) reported an increased risk of both pancreatic cancer and HCC in patients with DM (relative ratio, approximately 1.5) [20]. Subsequently, the association between DM and HCC has been observed in numerous cohort studies [21–24] and case-control studies [12–14, 25]. In 2006, a meta-analysis of 13 cohort studies and 13 case-control studies conducted by El-Serag et al. found that DM is associated with an approximately 2.5-fold increased risk of HCC [26]. In 2014, Tanaka et al. systematically reviewed epidemiologic investigations on DM and HCC among Japanese populations, 9 of the 10 relative risk (RR) estimates in the case-control studies and 17 of the 24 RR estimates in the cohort studies showed a weak to strong positive association between DM and HCC risk, indicating that the overall evidence in Japan strongly supports an increased risk of HCC among DM patients [27]. Other studies also reported a 2- to 3-fold increased risk of HCC in patients with DM [28], and this association was generally observed in patients free of viral hepatitis [13, 24, 29]. However, several studies conducted in Taiwan did not find an increased risk of HCC in patients with DM. In 2013, Chen et al. conducted a retrospective cohort study to explore risk factors for HCC in 56,231 adults and reported that DM, metabolic syndrome, and obesity were not risk factors for HCC, regardless of hepatitis B virus (HBV) or hepatitis C virus (HCV) status [30]. Likewise, a case-control study conducted by Lu et al. did not find an association between DM and HCC [31]. In a series of 823 HCC patients and 3459 controls, El-Serag et al. found that DM increased the risk of HCC only in the presence of other risk factors such as HBV, HCV, or alcoholic cirrhosis [32].

## 3. Biological Mechanisms Linking DM and HCC

The complex process of carcinogenesis can be divided into the following stages: initiation, promotion, and progression. Factors associated with the development of cancer may affect one or more stages. Although the precise biological mechanisms underlying the link between DM and HCC are not completely understood, the following factors may be involved in the neoplastic process: endogenous hyperinsulinemia (insulin resistance), exogenous hyperinsulinemia (treatment with insulin or secretagogues), hyperglycemia, and/or chronic inflammation.

### 3.1. Hyperinsulinemia, Insulin Resistance, and HCC

Elevated insulin levels caused by insulin resistance in fat, liver, and muscle tissue may explain, at least in part, the increased risk of HCC in DM patients [33]. Hyperinsulinemia can increase insulin-like growth factor 1 (IGF-1), which in turn can stimulate liver cell proliferation [34–41]. In addition, hyperinsulinemia could increase the secretion of matrix proteins and other precursors of hepatic fibrosis by hepatic stellate cells [42] and decrease mitochondrial *β*-oxidation of fatty acids [43], which is associated with hepatocellular injury, inflammation, and hepatic fibrosis. Furthermore, insulin resistance is independently associated with the progression of liver fibrosis, which is a risk factor for HCC.

### 3.2. Obesity, Hyperglycemia, and HCC

Obesity appears to be another factor linking DM and HCC. Type 2 diabetes mellitus (T2DM) is associated with central obesity, which promotes carcinogenesis through the secretion of proinflammatory cytokines by visceral adipose tissue [18]. Obesity is often associated with liver cirrhosis and liver fibrosis progression [44], a primary risk factor for HCC [45].

Hyperglycemia might also have carcinogenic potential [46] through the increased glycosylation of hemoglobin. Glycosylated hemoglobin releases iron, which is a powerful prooxidant that produces free radicals, thereby causing oxidative stress [36, 47]. The high serum iron levels associated with DM may also reflect increased body iron stores [36]. Thus oxidative stress may play a role in pathogenesis of DM and its complications, such as cirrhosis, which is a primary risk factor for HCC.

### 3.3. Synergistic Interactions between DM and Other HCC Risk Factors

Several studies have described synergistic interactions between DM and other HCC risk factors, such as viral hepatitis and heavy alcohol consumption [13, 48, 49]. Chen et al. investigated the relationship between DM and HBV/HCV infections in a cohort of 23,820 residents of Taiwan, who were followed for 14 years. The results showed that the combination of obesity and DM increased the risk of HCC more than 100-fold in HBV or HCV carriers [24]. Hassan et al. evaluated risk factors for HCC in a case-control study that included 115 HCC patients and 230 controls. The increased risk associated with the combination of heavy alcohol consumption and DM (odds ratio [OR], 9.9; 95% confidence interval [CI], 2.5–39.3) was higher than the risk associated with each risk factor alone [48]. These findings suggest that synergistic interactions between DM and other risk factors for HCC may play a role in hepatocarcinogenesis.

### 3.4. Inflammatory Cytokines, Epigenetic Mechanisms, and HCC

Chronic inflammation associated with DM may promote the development of HCC through the action of proinflammatory cytokines [50]. Several studies have observed elevated levels of tumor necrosis factor alpha (TNF-*α*) and interleukin-6 (IL-6) in obese individuals with DM [51–55]. These cytokines regulate the apoptotic regulators Bcl-2 and Bax, suggesting their potential as apoptotic and inflammatory markers for HCC [56–59]

Epigenetic modifications (DNA methylation, histone modification, and RNA interference) can activate proinflammatory genes that encode nuclear factor kappa B (NF-*κ*B) and proteins involved in JAK/STAT signaling, leading to the deregulation of metabolic pathways and hepatocellular transformation. Numerous epigenetic events and methylated genes have been detected in patient tissues or serum prior to or concurrent with a cancer diagnosis, suggesting their potential use as effective biomarkers for early detection [60–62].

## 4. Relationship between DM and HCC with Different Etiologies

Several studies have attempted to elucidate the relationship between DM and HCC with different etiologies. A retrospective analysis of patients in the US Department of Veterans Affairs database found that the risk of primary liver cancer was increased by DM only in patients with other risk factors such as HBV, HCV, or alcoholic cirrhosis [32]. However, subsequent studies reported the risk of HCC was increased with DM independent of alcoholic liver disease and viral hepatitis [63, 64]. We therefore reviewed studies exploring the relationships between DM and HCC with different etiologies [32, 65, 66].

### 4.1. Hepatitis C Virus

DM is closely associated with chronic HCV infection, which contributes to 25% of HCC cases globally [67]. Epidemiological studies have shown that DM is associated with a 2- to 3-fold increase in HCC risk in patients with chronic HCV infection, regardless of whether the patient has undergone curative hepatectomy or antiviral therapy [67–71]. A population-based study using data from the SEER-Medicare Linked Database also detected a 2- to 3-fold increased risk of HCC in chronic hepatitis C patients with DM [14]. Similarly, we previously demonstrated that DM confers a nearly 2-fold increase in the risk of HCC in treatment-naïve chronic hepatitis C patients in China [71], and a European study following 541 patients with chronic hepatitis C found that the incidence of HCC over 5 years was 11.4% for patients with DM and 5.0% for patients without DM [72]. However, in a prospective cohort of 54,979 subjects followed from 1999 and 2002 [23], T2DM increased the risk of HCC only in HCV-negative participants (RR, 2.08; 95% CI, 1.03–4.18). The* *reason* *for* *this* *discrepancy is unclear; however, the target population in this study had a relatively high rate of hepatitis infection, which was* *rare* *in* *most* *of* *the* *populations* *previously* *studied [73].

There are several other mechanisms that could account for the effect of DM on the risk of HCC in patients with chronic hepatitis C. First, insulin is a growth factor, and high insulin levels in patients with insulin resistance may interfere with the action of interferon, thereby decreasing both rapid and sustained virological responses [42, 74–76]. In addition, hyperglycemia [76, 77] may impair HCV eradication. Finally, fibrosis progresses more rapidly to cirrhosis in patients with insulin resistance, T2DM, and HCV, except for HCV genotype 3, which is less responsive to interferon treatment.

### 4.2. Hepatitis B Virus

HBV is a hepatocarcinogenic virus that infects 400 million people globally and accounts for approximately 54% of HCC cases worldwide [78, 79] and 85% of the HCC cases in China [80–82]. The relationship between DM and HBV-related HCC remains unclear. A long-term community-based cohort study in Taiwan reported a 2- to 3-fold higher risk of HCC in patients with DM who were also HBV-positive (adjusted RR, 2.27; 95% CI, 1.10–4.66) [24]. Gao et al. found that DM is an independent risk factor for cirrhosis progression to HCC in patients with simple HBV infection [83], and Hsiang et al. found that T2DM was predictor of liver-related complications and HCC in patients with HBV cirrhosis [84]. A Japanese study of 156 HCC patients with chronic HBV infection also suggested the involvement of T2DM in hepatocarcinogenesis in HBV-positive patients [85]. In 2012, a case-control study conducted in Taiwan concluded that synergistic interactions between T2DM and HBV infection increased the risk of HCC [86]. In 2015, a cohort study using data from the Taiwanese National Health Insurance Research Database reported that new-onset DM was associated with an increased risk of HCC in HBV-positive patients (RR, 1.628; 95% CI, 1.114–2.378) [87]. However, a recent study by Han et al. did not find that T2DM increased the risk of HCC in patients with HBV-related cirrhosis [88]. Similarly, a 2013 a cross-sectional case-control study of 122 HBV-infected cirrhotic patients with HCC and 248 cirrhotic patients without HCC reported that DM was not a significant risk factor for HCC [30].

Few studies have investigated potential mechanisms underlying this association between DM and HBV-related HCC. In 2005, a community-based study of 3587 HBV-positive participants found that high HBV load was inversely associated with extreme and central obesity in HBeAg-seropositive patients (adjusted OR, 0.17 and 0.44, resp.; 95% CI, 0.05–0.63 and 0.25–0.78, resp.) [89]. However, HBV load was not associated with liver steatosis in HBeAg-seropositive or HBeAg-seronegative patients (adjusted OR, 1.46 and 0.88, resp.; 95% CI, 0.90–2.36 and 0.72–1.08, resp.). These findings suggest that obesity may cause liver damage via oxidative stress and hepatic steatosis independently of HBV infection.

### 4.3. Nonalcoholic Fatty Liver Disease

Nonalcoholic fatty liver disease (NAFLD) ranges from simple steatosis, nonalcoholic steatohepatitis (NASH) characterized by inflammation, and NASH-related fibrosis leading to cirrhosis. NAFLD is now the leading cause of chronic liver disease (including cryptogenic cirrhosis) in both developed and developing countries with rising obesity rates [45, 89–92]. NASH has been shown to lead to cryptogenic HCC [93–95], and HCC tumors related to NAFLD tend to be larger and more advanced when detected compared with those related to hepatitis virus infection [96, 97].

Although DM is involved in the development of HCC in NAFLD [98], it is difficult to study causality because the risk factor (DM) is affected by and interacts with the outcomes (NAFLD and HCC) [99]. Numerous case reports and case reviews indicate DM appears to be a risk factor for NASH, which is a cause of cryptogenic HCC [50], and DM is an independent risk factor for HCC in patients with NASH [100–103]. In addition, obesity and DM are associated with liver fibrosis severity in patients with NASH [45].

### 4.4. Alcohol

Alcohol abuse is a common cause of HCC, especially in western countries. A study by Kikuchi et al. of 1,478 alcoholic liver cirrhosis patients identified DM as a risk factor for HCC in this patient population [104]. Similarly, a cohort study conducted by Raff et al. found that DM increased the risk for cirrhosis and HCC in patients with alcoholic liver disease [105].

Alcoholic liver disease and NAFLD have similar pathogenic mechanisms and histological findings but different phenotypes and risk factors [106], with a histological spectrum that ranges from simple steatosis to steatohepatitis, fibrosis, cirrhosis, or HCC. DM, which is a risk factor for NAFLD, may also exacerbate alcoholic liver disease [107] and promote alcohol-related HCC. In fact, a synergistic interaction between alcohol consumption and DM has been observed [13, 48], and alcohol-induced oxidative stress in patients with DM may promote cirrhosis, DNA damage, and ultimately HCC [108, 109].

## 5. Antidiabetic Medication and Risk of HCC

Results of in vitro and in vivo preclinical studies have suggested that antidiabetic drugs influence the development of multiple cancers. Epidemiological evidence indicates that metformin and thiazolidinediones (TZDs) are associated with a lower overall cancer incidence [17, 110–113]. However, insulin and insulin secretagogues are associated with higher cancer incidence and cancer-related mortality [40, 114]. In this section, we reviewed the effect of conventional antidiabetic drugs on the risk of HCC in patients with DM.

### 5.1. Metformin

The metformin is a first-line therapy for T2DM and is often prescribed for prediabetes and DM that is less severe or of shorter duration. Metformin can decrease blood glucose and insulin levels in these patients; however, the mechanism underlying this effect is not entirely clear [115]. In addition, results of several pharmacoepidemiologic studies suggest that metformin use lowers the incidence of cancers, including HCC, in patients with DM [17, 113], whose risk of developing HCC is at least 2-3 times higher than individuals without DM [14]. Long-term metformin treatment appears to inhibit hepatocellular transformation, decreasing the risk of HCC to levels similar to that of nondiabetic patients [116–122]. In a rat model of HCC, DePeralta et al. found that HCC incidence was decreased by 44% when metformin treatment was initiated at the first signs of fibrosis but was unchanged when metformin was not initiated until the first signs of cirrhosis [123]. A nationwide case-control study conducted by Chen et al. [120] found that metformin decreases the risk of HCC in patients with DM by 7% with each additional year of use. However, a cohort study conducted in the UK did not find a lower incidence of HCC in patients receiving metformin compared with those receiving sulfonylurea [124]. Furthermore, a meta-analysis of randomized controlled trials comparing the risk of cancer in patients receiving metformin or other antidiabetic drugs did not observe a protective effect of metformin against HCC [125].

The molecular mechanism underlying the antitumor activity of metformin is unclear. Results of studies in breast cancer cells suggest that metformin inhibits cancer by activating AMP-activated protein kinase (AMPK), which may lead to growth inhibition, thereby decreasing protein synthesis [126]. Other studies indicate that metformin inhibits tumorigenesis through both insulin-dependent and insulin-independent mechanisms [127] and that its effects may be lower in patients with lower insulin levels.

In insulin-resistant ob/ob mice, metformin improves fatty liver disease, possibly by attenuating hepatic expression of TNF-*α*, which promotes insulin resistance and plays a role in hepatocarcinogenesis [128]. Results of several small-scale trials demonstrate the potential for metformin to improve liver histology and body weight in patients with NAFLD [129–131], suggesting another potential pathway by which metformin may prevent HCC. However, a more recent study did not observe a significant improvement in liver histology in NAFLD patients receiving metformin [132]; thus further research evaluating liver histology in patients receiving metformin is needed.

### 5.2. Thiazolidinediones

Thiazolidinediones (TZDs) are peroxisome proliferator-activated receptor gamma (PPAR*γ*) agonists that lower insulin resistance without directly affecting insulin secretion. Pioglitazone and rosiglitazone are the two TZDs currently available in the China.

Although the risk of HCC in patients using TZDs is unclear [133], results of several studies indicate that TZDs may exert a beneficial effect [112, 134–138]. A case-control study by Chang et al. evaluated the effect of TZDs on the risk of liver cancer by identifying 10,741 patients with both DM and liver cancer and 70,559 patients with DM only in the Taiwan National Health Insurance claims database. Their results indicated that both pioglitazone and rosiglitazone significantly decreased the risk of liver cancer [112], with greater benefits associated with higher cumulative dosage [112]. Using the same database, Chen et al. [120] showed that each additional year of TZDs use decreased the risk of HCC in diabetic patients by 9%. In contrast, a nested case-control study using healthcare utilization databases in Italy did not detect a significant effect of TZDs on the risk of HCC [122]. Similarly, a meta-analysis of four observational studies did not find a significant decrease in the risk of HCC in patients with DM who received TZDs (adjusted OR, 0.5; 95% CI, 0.28–1.02) [120, 134, 139, 140].

Anticancer activities of TZDs observed in vitro include growth inhibition and promotion of apoptosis and cell differentiation [141, 142]; thus PPAR*γ* has been identified as a potential therapeutic target for chemoprevention and cancer therapy [143, 144]. However, recent studies have reported that the effects of TZDs on cell growth do not require the presence of PPAR*γ* [145–147]. Furthermore, PPAR agonists appear to promote tumorigenesis in multiple rodent species and in both sexes [148], suggesting that TZDs may increase the risk of cancer or promote cancer progression in humans.

### 5.3. Insulin Secretagogues, Insulin, and Insulin Analogs

Secretagogues bind to specific cell receptors on *β*-cells, leading to depolarization of the plasma membrane and release of insulin stores. This class of drugs includes sulfonylureas and rapid-acting glinides. Results of observational studies indicate that secretagogues increase the risk of HCC in patients with DM [112, 116, 121, 122, 134]. Hassan et al. found that sulfonylurea use increased the risk of HCC in diabetic patients by 7-fold (adjusted OR, 7.1; 95% CI, 2.9–16.9) [134]. Bosetti et al. reported a higher risk for repaglinide (OR, 2.12; 95% CI, 1.38–3.26) than for sulfonylureas (OR, 1.39; 95% CI 0.98–1.99) [122]. However, in most of these studies, few of the diabetic patients using sulfonylureas developed cancer [149, 150].

Many patients with DM eventually require insulin as *β*-cell function decreases; therefore, insulin is used more often by patients with longer duration of DM and those with more complications [133, 151, 152]. Yu et al. reported a higher risk of HCC in patients with T2DM treated with insulin (RR, 18.5; 95% CI, 2.2–156.0) [108], and a meta-analysis performed by Singh et al. reported a 161% increased risk of HCC in patients treated with insulin [121]. Bosetti et al. reported that the risk of HCC was further increased with prolonged insulin use [122]. However, studies by Miele et al. did not find a significantly higher risk of HCC in patients receiving insulin [134, 153].

Both insulin and insulin secretagogues upregulate IGF-1 activity, which increases hepatic cell proliferation and alters cell metabolism [40, 154, 155]. In addition, these drugs can cause hyperinsulinemia, hepatotoxicity, and weight gain [156], indicating that their use in patients with chronic liver disease may increase the risk of HCC [157]. Future studies comparing the effects of antidiabetic medications on HCC risk should consider the severity of DM, because insulin is often prescribed in DM that is more severe or of longer duration.

## 6. Duration of DM and HCC Development

The duration of DM prior to HCC development may play an important role in the relationship between DM and HCC. A case-control study in Canada showed that the risk of HCC was higher in individuals with a longer history of DM [28]. Similarly, a meta-analysis by Wang et al. indicated that those with a history of DM > 10 years had the highest risk of HCC; however, this study had relatively low power because of the small number of studies included [158]. Hassan et al. reported that, compared with patients with a DM duration of 2–5 years, the risk of HCC was higher in those with a DM duration of 6–10 years (adjusted OR, 1.8; 95% CI, 0.8–4.1) or >10 years (adjusted OR, 2.2; 95% CI, 1.2–4.8) [134]. Miele et al. also found a higher risk of HCC with longer duration of DM (OR, 2.96 for <10 years; OR, 5.33 for ≥10 years) [153]. Several other studies also observed that longer duration of DM was associated with an increased risk of HCC [64, 108, 138, 159–161]. In contrast, Wang et al. found that the risk of HCC among patients with a duration of DM < 5 years was higher than that of patients with duration of 5–10 years (RR, 3.76 versus 3.17) [159]; however, this difference was not significant. In our previous study, results of multivariate analysis showed that longer duration of DM (>5 years) did not significantly increase HCC risk [71].

Taken together, most of studies which investigated positively relationship between DM duration and HCC risk suggest that duration of DM > 10 years increases the risk of HCC; however, larger studies are needed to confirm these results. Meanwhile, the severity of DM should be clarified because patients with longer duration are likely to have more severe DM which might impact HCC development.

## 7. Conclusion

Diabetes mellitus is globally endemic, and increasing evidence from observational studies suggests that DM is a risk factor for HCC. Therefore, the increasing prevalence of DM may increase the incidence of HCC. The use of metformin, first-line therapy for DM, is associated with a decreased incidence of HCC, whereas insulin, generally used by patients with longer duration of DM or more complications, is associated with an increased incidence of HCC. Further research is needed to determine whether these relationships are causal or influenced by the duration or severity of DM and whether the results were affected by residual bias or misclassification.

In addition, studies are needed to elucidate the possible effects of antidiabetic drug type/dosage and duration of DM on the risk of HCC and to better understand the relationship between DM and HCC with different etiologies.
